# Projections of the future disappearance of the Quelccaya Ice Cap in the Central Andes

**DOI:** 10.1038/s41598-018-33698-z

**Published:** 2018-10-22

**Authors:** Christian Yarleque, Mathias Vuille, Douglas R. Hardy, Oliver Elison Timm, Jorge De la Cruz, Hugo Ramos, Antoine Rabatel

**Affiliations:** 1 Department of Atmospheric and Environmental Sciences, University at Albany, Albany, New York 12222 USA; 2 Department of Geosciences, University of Massachusetts Amherst, Massachusetts, USA; 3 Integración de Sistemas Social-Ambiental e Impacto (ISSAI) S.A.C., Lima, Peru; 4 Facultad Agronomía, Universidad Nacional Agraria La Molina (UNALM)., Lima, Peru; 5 Université Grenoble Alpes, CNRS, IRD, Grenoble-INP, Institut des Géosciences de l’Environnement (IGE, UMR 5001), Grenoble, 38000 France

## Abstract

We analyze the future state of Quelccaya Ice Cap (QIC), the world’s largest tropical ice cap with a summit elevation of 5680 m a.s.l., which, in terms of its elevation range (~5300–5680 m a.s.l.), is representative of many low-elevation glacierized sites in the tropical Andes. CMIP5 model projections of air temperature (Ta) at QIC indicate a warming of about 2.4 °C and 5.4 °C (respectively) for RCP4.5 and RCP8.5 scenarios by the end of the 21^st^ century, resulting in a pronounced increase in freezing level height (FLH). The impact of this warming on the QIC was quantified using equilibrium-line altitude (ELA) projections. The change in the ELA was quantified based on an empirical ELA–FLH relationship, and calibrated with observations of the highest annual snowline altitude (SLA) derived from LANDSAT data. Results show that from the mid-2050s onwards, the ELA will be located above the QIC summit in the RCP8.5 scenario. At that time, surface mass balance at QIC and most tropical glaciers at similar elevations will become increasingly negative, leading to their eventual complete disappearance. Our analysis further corroborates that elevation-dependent warming (EDW) contributes significantly to the enhanced warming over the QIC, and that EDW at Quelccaya depends on the rate of anthropogenic forcing.

## Introduction

**Figure 1:**
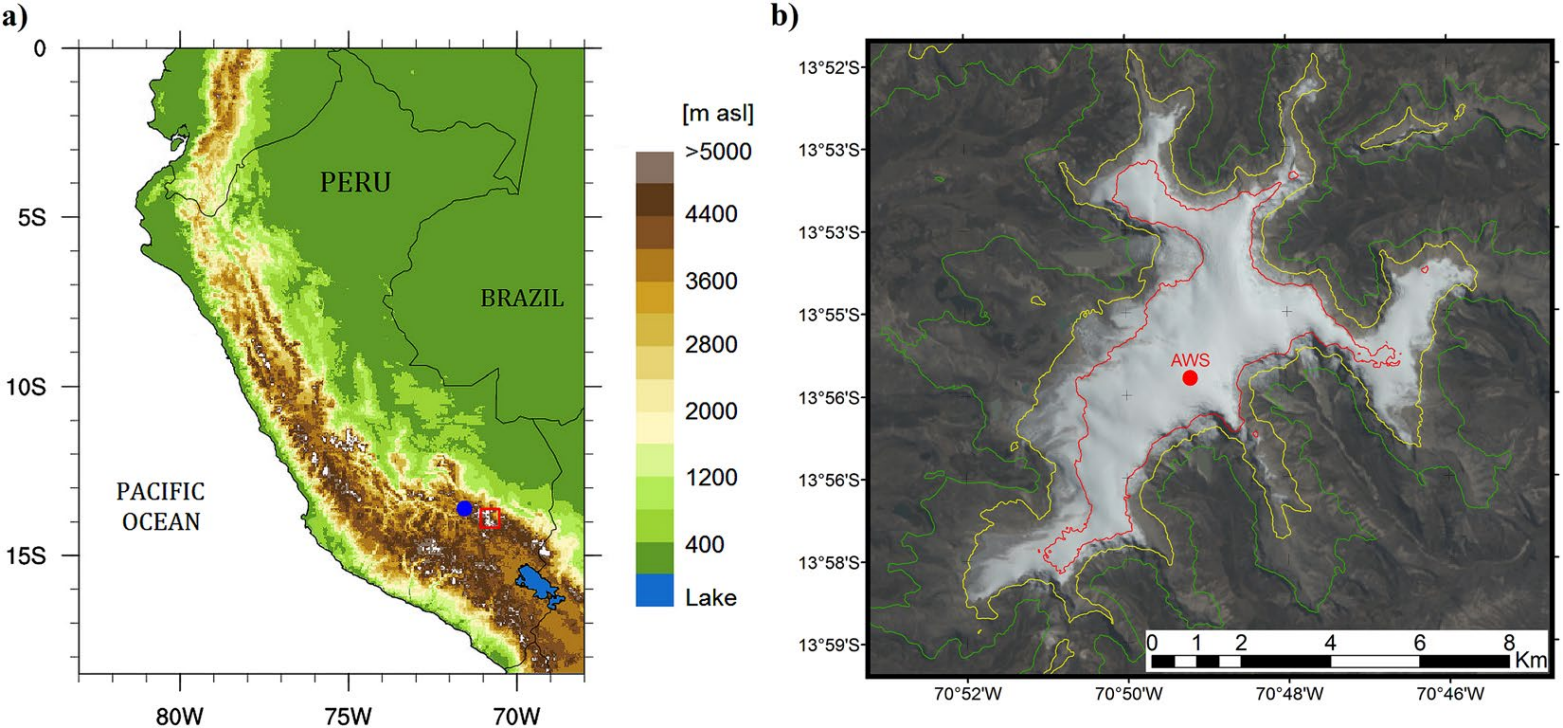
Location of Quelccaya ice cap in the Peruvian Andes. (**a**) Central Andes topography (color shading), and locations of QIC (red square marker) and Ccatcca station (blue dot). (**b**) LANDSAT 8 image (bands 4,3,2/RGB) of QIC on 2^nd^ August 2017. The AWS location is shown with a red dot. The color contours represent the 5100 (green), 5300 (yellow), and the 5500 m a.s.l. (red) isolines.

A more thorough understanding of future glacier changes in the tropical Andes is critical, given their prominent role in dry season water supply, ecosystem services, and impacts on tourism, natural hazards and cultural values and belief systems of local populations^1^. About 99% of the world’s tropical glaciers are located in the Andes, with Peru alone containing about 70% of them^2–4^. Quelccaya ice cap (QIC) is located in the Cordillera Vilcanota of southern Peru (13°56’S, 70°50′W, Fig. 1). With a median area of about 50.2 km^2^ over the 1975–2010 period^5^, QIC is the largest tropical ice cap. The average elevation of the ice margin is ~5300 m above sea level (m a.s.l.) and the approximate summit elevation is 5680 m a.s.l.; therefore, QIC is representative of many tropical glaciers in the Andes with a relatively low summit elevation^6–8^. In comparison, the lowermost elevations reached by the largest glaciers in the tropical Andes is typically close to 4850–4900 m a.s.l., whereas their upper reaches are frequently above 6000 m a.s.l. (the highest elevation being reached at the peak of Mount Huascaran at 6768 m a.s.l. in the Peruvian Cordillera Blanca).

The extent of the QIC has been affected by the increase in Andean surface temperature^9,10^, but potentially also by variations in precipitation^4,11^. The El Niño - Southern Oscillation (ENSO)^8^, the South American Summer Monsoon (SASM)^12^, and cold air incursions from the extratropics^13^ also affect QIC conditions on an interannual time scale. However, no continuous surface mass balance and ice dynamics measurements exist on QIC; hence the relationship between the reduction in surface area and loss of glacier mass is not known.

Although precipitation is an important variable affecting glacier surface mass balance, observational studies document that no significant changes in precipitation occurred in this region during the past five decades^14–16^. Air temperature on the other hand has been increasing over the Peruvian Andes over the last six decades^9,11,14^, in agreement with the regional increase in temperature over the entire tropical and sub-tropical Andes^4^. The increasing temperature is a combined effect of natural multi-decadal variability (i.e. the Pacific Decadal Oscillation) and anthropogenic radiative forcing^10^. Due to this warming, QIC is retreating at an accelerated pace, with a shrinking of the QIC area at a rate of 0.57 ± 0.10 km^2^ yr^−1^ over the 1980–2010 period^5^. This retreat is consistent with the reduction in glacierized surface area observed throughout the tropical Andes, including in the Cordillera Blanca and the Cordillera Ampato^3,4^, located to the north and south of the Cordillera Vilcanota and QIC, respectively.

Model projections of twenty-first century climate change indicate a substantial future temperature increase across the central Andes, ranging between +3 and +5 °C depending on region, model and emission scenario^17–19^. It is important to note that the rate of warming tends to be further amplified with elevation in many mountain regions due to elevation-dependent feedbacks^20,21^. Given that coarse global models do not adequately resolve the Andean topography, this effect is likely underestimated in surface temperature estimates from global models^22^, but likely less so when considering the free tropospheric temperature trends^17^. This elevation-dependent warming (EDW) has been documented over the tropical Andes, both in modern observations and future model scenarios^10,21,23^.

A fairly simple diagnostic that can be calculated from reanalysis and model data, and is more relevant for glacier mass balance than surface temperature, is the freezing level height (FLH). Increasing FLH in the Central Andes negatively affects the surface mass balance of glaciers, by changing the rain/snow ratio and increasingly exposing lower reaches of glaciers to rain as opposed to snow^4^. Hence a rise in the FLH does not only directly affect the glacier surface mass balance through higher temperatures, leading to more melt, but also impacts accumulation and glacier surface albedo^24^. The FLH increased by approximately 160 m over the last five and a half decades over the Cordillera Blanca and Cordillera Real^4^, located to the north and south of QIC, respectively. The mean annual FLH in the Cordillera Vilcanota was 5010 m a.s.l. over the 1980–2015 period^25^, with a higher FLH during the warmer wet season and a lower FLH during the slightly colder dry season^4^, respectively. Historically the increase in the FLH in the tropics can be empirically described as a linear response to the increase in tropical sea surface temperature (SST)^26,27^. Moreover, the FLH over this region is dependent on the phase of ENSO and responds to both interannual and decadal-scale changes in tropical Pacific SST^9,28^.

While both anthropogenic and natural forcings may affect glacier surface mass balance variability on QIC on an interannual timescale^7,10^, the accelerated rate of retreat observed over the last decades^5^ is consistent with the gradual disappearance of lower-lying Andean glaciers as is being observed for example in Bolivia^29^, Colombia^30^ and Venezuela^31^. Modeling studies suggest continued future shrinkage of tropical Andean glaciers, with some completely disappearing by the end of the 21^st^ century^1,25,32^, thereby significantly reducing dry season runoff^33–36^.

Here we assess the rate of change of surface air temperature and FLH over QIC, using CMIP5 projections based on two different emission scenarios. In contrast to variables related to the hydrologic cycle (e.g., precipitation), free-air temperature is quite accurately simulated by GCM’s, and very well represented by reanalysis^37^. Surface temperature is also well reproduced by most GCM’s, although there is a substantial warm bias over the Andes due to the low topography in the models. Here we rely on *in-situ* air temperature data recorded by an automated weather station (AWS) at the summit of QIC^9,13^ to remove the temperature bias from both reanalysis and GCM output, allowing for an accurate future projection of changes in FLH. We further take advantage of the documented close empirical relationship between FLH and the glacier equilibrium-line altitude (ELA) on tropical Andean glaciers^1^ to project the future rise of the ELA on QIC under various emission scenarios. Although no spatially-comprehensive ELA measurements exist on QIC, the ELA can be constrained by determining the snowline during the dry season using satellite data^38^. Hence the aim of this study is to determine how imminent a future disappearance of the QIC really is, and to what extent the timing depends on the emission scenario. We also consider the influence of EDW on the rate of the ELA rise, by comparing CMIP5 simulations with an empirical model that relates tropical SST to FLH assuming a constant lapse rate^9^.

## Data and Methods

### Observational data

Daily mean non-aspirated temperature and snow height data between 21-07-2004 and 22-07-2017 from an AWS installed at QIC summit (5680 m a.s.l., 13.93°S, 70.82 W) were used to bias-correct air temperature from reanalysis and CMIP5 models, and to inform LANDSAT image selection (see below). As reference, the mean annual air temperature (Ta) at QIC from this dataset is −3.99 °C over the 2005–2016 period. Additionally, daily rainfall data, available over the period 1979–2016 from Ccatcca station (3693 m a.s.l., 13.61°S, 71.5603°W, closest station to QIC, Fig. 1a), maintained by the Peruvian National Meteorological and Hydrological Service (*Servicio Nacional de Meteorología e Hidrología del Perú*, SENAMHI), were used for the LANDSAT image-selection process. Finally, monthly mean SST data from the NOAA Extended Reconstructed Sea Surface Temperature v5 (ERSST) dataset^39^ were extracted over the tropical belt (28.5°S–28.5°N) from 1950 to 2017. Anomalies were calculated using 1979–2005 as the reference period, and then spatially averaged to obtain a tropical SST anomaly (SSTA) time series.

### Ta and FLH calculation from reanalysis products

Several studies have shown that mid- and upper-tropospheric temperatures are fairly accurately reproduced by GCM’s and reanalysis products over the central Andes^9,22,37^. In the present study, we relied on monthly ERA-interim reanalysis^40^, covering the 1979–2017 period, since this dataset has higher skill in reproducing observed temperature variability over the central Andes region compared with other reanalyses^37^. Monthly Ta at the elevation of QIC summit was calculated by interpolating Ta and geopotential height (Zg) between 400 and 500 hPa pressure levels, which are the nearest standard pressure levels above QIC summit. The ERA-interim products were resampled to a 2.5° grid resolution, mimicking the spatial scale of the majority of CMIP5 models. A bias correction was applied to the reanalysis Ta using as reference the observed data from the AWS at QIC summit. Similarly, the FLH at QIC was calculated as the elevation of the 0 °C isotherm using a linear interpolation of ERA-interim Zg and (bias-corrected) Ta between 500 and 600 hPa. The same approach was applied to Ta and FLH from CMIP5 simulations to remove temperature biases from the simulations and to calculate historical and future FLH as simulated by the models.

The FLH in the tropics can be estimated using an empirical linear relationship with tropical SST^9,26–28^. Here, we followed this approach by comparing the QIC FLH derived from ERA-interim with ERSST data over the tropics for the period 1979–2017. This linear SST-FLH relationship was then applied to tropical SST simulated with CMIP5 models from both historic runs and future projections. Comparing the FLH at the elevation of QIC as simulated by the CMIP5 models (henceforth labeled FLH_atm_) with the FLH estimated from a linear empirical dependency with SST (henceforth labeled FLH_SST_) yields an estimate of future EDW, since the lapse rate is allowed to adjust to EDW feedbacks in the coupled CMIP5 simulations used to determine FLH_atm_^10,21,23^, but held fixed at observed present-day values in the latter empirical approach of calculating FLH_SST_. A similar approach is often applied in paleoclimate assessments of how the tropical lapse rate has changed, in order to reconcile tropical snowline reconstructions with estimates of past changes in tropical SST^41,42^. It is noted that the linear regression model is fitted with observed SSTA, hence CMIP5 model biases are implicitly removed.

### Snowline altitude derived from satellite data

Since no long-term surface mass balance measurements exist from QIC, we applied an indirect method to determine the ELA^38^. This method is based on the fact that on glaciers in the outer tropics the highest snowline altitude (SLA) reached during the dry season is representative of the annual ELA of the same hydrological year. Here, the annual SLA was determined using data from LANDSAT-5, -7 and/or -8 with 30 m spatial resolution between 1992 and 2017, selecting one image (or date) per year (Table 1). The selection criteria for the LANDSAT data consist of choosing the date with the highest SLA, avoiding dates with recent snowfall and rainfall events. As indicated in Table 1 the chosen LANDSAT images date to the dry season and early transition season, i.e. from June to October. Images that postdate recent snowfall on QIC were flagged based on the daily snow height time series from the AWS at QIC, and daily rainfall data from Ccatcca station. Additionally, the ALOS PALSAR^43^ digital elevation model (DEM) with sensor FBS, path 101, 12.5 m^2^ cell size, WGS 1984 UTM zone 19 S projection, from 26 Oct. 2007 was used to determine the elevation of the SLA pixels in the LANDSAT data. The spatial resolution of PALSAR is superior to conventional products, and it includes terrain, radiometric and orthorectification corrections.

**Table 1:** LANDSAT sensor and selected date for obtaining annual snowline altitude (SLA), 1992–2017.

Sensor	Date	Sensor	Date
LT5	10 Jun 1992	LT5	17 Aug 2005
LT5	29 Jun 1993	LE7	15 Oct 2006
LT5	18 Jun 1994	LT5	23 Aug 2007
LT5	07 Sep 1995	LE7	02 Sep 2008
LT5	07 Jul 1996	LT5	15 Oct 2009
LT5	27 Aug 1997	LT5	16 Sep 2010
LT5	15 Sep 1998	LT5	18 Aug 2011
LE7	10 Sep 1999	LE7	31 Oct 2012
LT5	03 Aug 2000	LC8	22 Jul 2013
LT5	06 Aug 2001	LC8	13 Oct 2014
LE7	04 Oct 2002	LC8	14 Sep 2015
LT5	29 Sep 2003	LC8	16 Sep 2016
LE7	19 Jun 2004	LC8	05 Oct 2017

**Figure 2:**
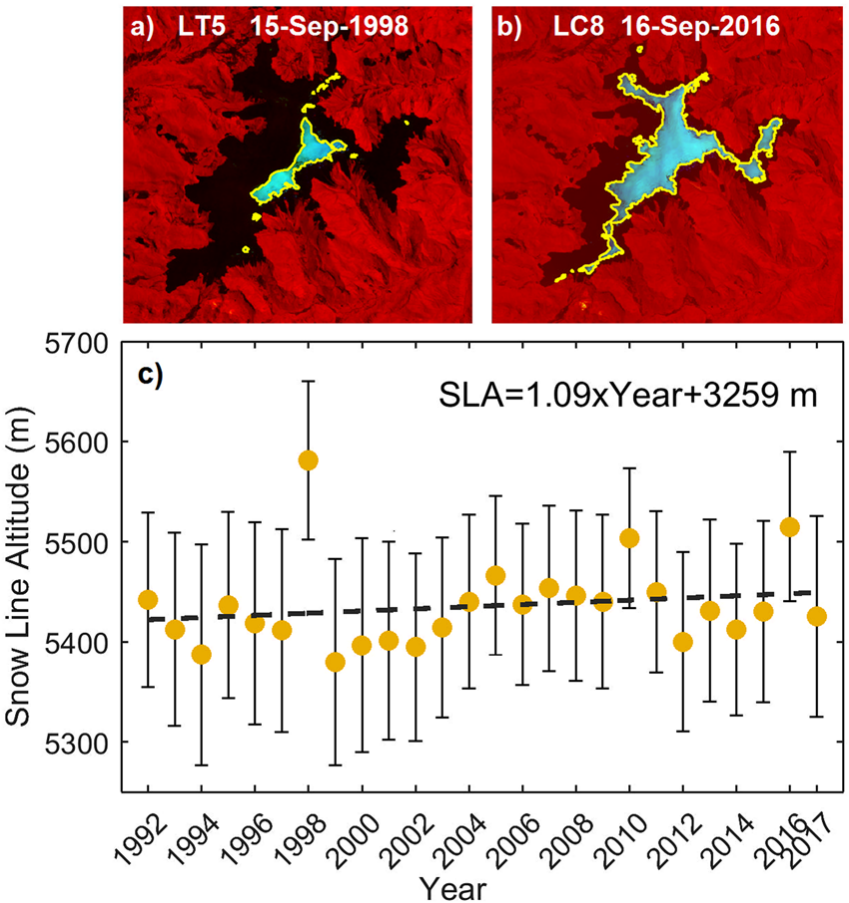
Snowline altitude using LANDSAT images. (**a**) Snow region in light blue, as a RGB composite using LANDSAT 5 (LT5) images of bands 5, 4, and 2, having applied histogram threshold values of 155 and 174 for bands 4 and 2, respectively. Snow line (SL) perimeter is shown in yellow and the black shading indicates the spatial domain of QIC, on 15 Sept. 1998. The mean SLA is calculated as the average elevation of all DEM cells coinciding with the location of the snow line perimeter. (**b**) As in (**a**), but for the date selected in 2016, and bands 6, 5 and 3 from LANDSAT 8 (LC8), with histogram threshold values of 99 and 117 for bands 5 and 3, respectively. (**c**) Mean SLA obtained each year (i.e. mean elevation corresponding to yellow perimeter). The dashed line represents the linear trend with equation indicated in the legend. The non-zero trend was verified using an F-test (p-value < 0.001), both with and without outliers (strong El Niño years 1998, 2010, 2016) included. Whiskers are representing the SLA error associated with the SLA standard deviation, LANDSAT spatial resolution, DEM vertical accuracy and slope in the SL perimeter.

We display the LANDSAT data as RGB images using Shortwave Infrared 1 (SWIR), Near Infrared (NIR) and Green bands^38^. A threshold was set to detect snow areas in NIR and Green bands^38^ since lighting conditions vary through dates (e.g., Fig. 2a,b). For the images listed in Table 1, thresholds between 80 and 180 were used in the histogram from NIR and Green bands. Finally, the perimeter of the snow-covered area above the SLA was hand-digitized and projected on the DEM to extract the corresponding elevation values of the SLA. A mean SLA was calculated for each date by averaging the elevation corresponding to all SLA pixels.

Although the SLA delineation is often unambiguous, it is worth noting that the determination is threshold-selection sensitive. Measurement errors can also be produced by debris located on the lower slopes of the ice cap. Moreover, the western side of the QIC has a higher sensitivity (and hence a lower uncertainty) in SLA detection than the eastern side, since the western slope extends over flatter terrain covering a larger surface area per unit elevation change. In general, however, these errors introduced by complex topography, insolation or debris-covered ice are reduced when the SLA is measured at the end of the dry season, as the SLA reaches its highest position. At that time of year the terrain is more symmetric and uniformly sloped on western and eastern QIC sides, as observed from vertical profiles^44^ and surface area^5^ measurements. Errors in hand-digitized SLA at QIC are comparable with those obtained through automated techniques^5^, although some studies use hand-digitized SLA as a reference or true value, due to the scarcity of *in situ* data^45^. Studies determining QIC surface area estimated the hand-digitized values to be 99.8% accurate^45^, while using an automated technique^5^ resulted in an estimated uncertainty in their areal measurements of 5%. For the current study, the mean SLA is obtained as the mean elevation of all cells corresponding to SLA pixels, along the entire ice cap perimeter (yellow perimeter in Fig. 2a,b), which corresponds to approximately 800 to 1200 cells, depending on the year. The resulting mean SLA per year (or date selected) is plotted in Fig. 2c. The SLA error is associated with a) the standard deviation of the estimated mean SLA per year. It ranges between 64.2 m and 102.6 m. This standard deviation value is higher than the values mentioned in other similar studies; this is most likely due to exposure effects around the ice cap, b) their corresponding slopes [range between 15.3° and 27.1°], c) the 30 m LANDSAT spatial resolution (considering a ± 1 pixel deviation range, the final dispersion value was taken as 90 m), and, d) the vertical accuracy of the DEM calculated as the mean of the differences between the ‘true’ elevation measured at 53 locations distributed along a transect from bedrock below the ice cap up to the summit of QIC in 2013, and their corresponding DEM values (2007). The error propagation method was used for the calculation of the SLA error^46^ resulting in total errors between 70 and 110 m. The annual SLA uncertainty is plotted as ± 1 standard error represented by the whiskers in Fig. 2c. It is worth noting that the SLA distribution per date selected has an approximately Gaussian distribution, and the 95% confidence intervals calculated with t-distribution presented values similar to the ones calculated using bootstrap analysis. The non-zero trend presented in Fig. 2c is statistically significant (F-test, p-value < 0.001), regardless of whether the three outlier years associated with strong El Niño years (1998, 2010, 2016) are included or not.

An error source which is more difficult to account for is the combined effect of an intermittent image acquisition schedule and scene obscuration by clouds. For example, LANDSAT 7 provides an image every 16 days. When LANDSAT 8 went into service with an offset orbit, 8-day repeat coverage became available. However, sometimes cloud cover obscures the glacier and hence the SLA cannot be mapped from every available image. The combined effect of these two issues is that our remotely-sensed, highest-annual SLA determination will always be lower than or equal to the actual annual SLA. Our observations in recent years, when AWS measurements and additional imagery are also available (e.g., ESA’s Sentinel-2) provide some assurance that this error is not large, as the SLA reflects seasonal snowfall as well as dry-season weather.

### Derivation of the ELA-FLH relationship

The SLA determined in the previous step can be considered a reasonable proxy for the ELA in each year^38^. On the other hand, to project the future change in the ELA on QIC over the course of the 21^st^ century, we take advantage of the close linear relationship between ELA and FLH on tropical Andean glaciers^1^. We first calculated FLH over QIC using bias-corrected reanalysis data over the period of overlap with satellite data. The FLH is calculated as the average of the hydrologic year, which runs from Sep. to Aug. Finally, projected future changes in the ELA were calculated by applying the present day FLH-ELA relationship to future FLH simulated by bias-corrected CMIP5 models, as outlined in the Data and Methods section.

### GCM data

16 CMIP5 models^47^ (CMCC-CMS, CNRM-CM5, GFDL-CM3, GFDL-ESM2G, GFDL-ESM2M, GISS-E2-R, HadGEM2-AO, HadGEM2-CC, IPSL-CM5A-MR, MRI-CGCM3, MPI-ESM-LR, MPI-ESM-MR, MIROC-ESM, MIROC5, NorESM1-M, NorESM1-ME) for historical (1950–2005), RCP4.5 and RCP8.5 scenarios (2006–2100) were selected for our analysis. The three variables of interest include Ta, Zg, and SST. From those variables we calculated the FLH_atm_ and FLH_SST_ and their corresponding ELA_atm_ and ELA_SST_ projections in the same way as previously described for the reanalysis products, using the model or reanalysis grid cell encompassing QIC.

## Results

### Future projections of air temperature and FLH over QIC

**Figure 3:**
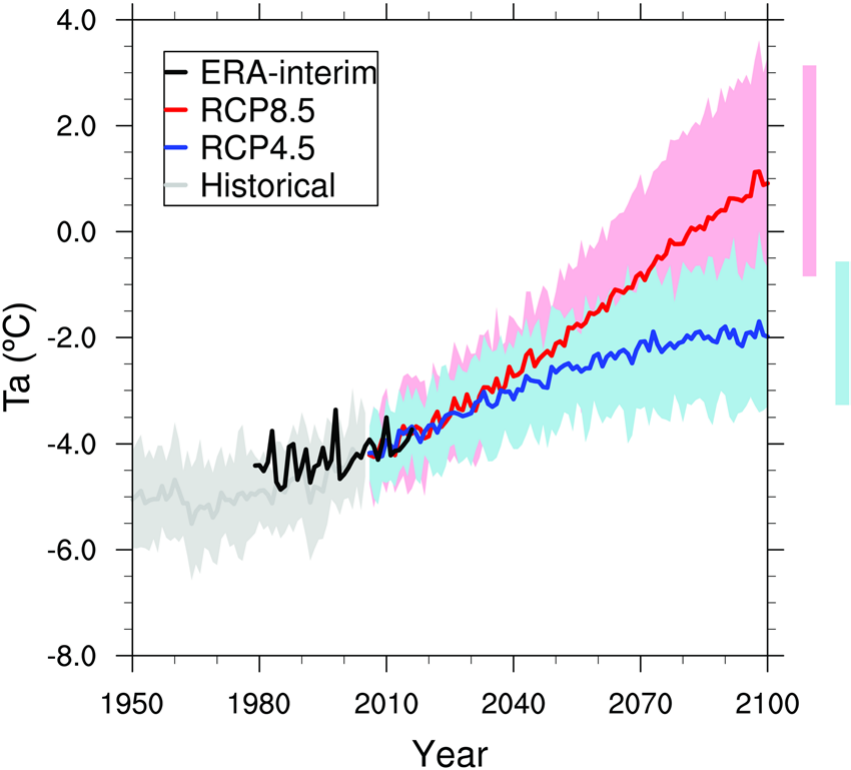
Annual air temperature projections at QIC summit. Annual mean air temperature (Ta) from 2.5° ERA-interim reanalysis (thick black line, 1979–2016), historical (gray, 1950–2005) and future (2006–2100) RCP4.5 (blue) and RCP8.5 (red) simulations. Ta at QIC summit elevation was calculated using the lapse rate between 400 and 500 hPa levels. Each data set was bias-corrected with AWS temperature data (2005–2016) from QIC summit. Thick lines represent the historical, RCP4.5 and RCP8.5 ensembles of 16 CMIP5 models and shading represents the 95% confidence interval. Likely ranges for QIC’s air temperature projections by the end of the 21st century are indicated by vertical bars.

Figure 3 shows the annual Ta at QIC from 2.5° ERA-interim reanalysis and CMIP5 simulations calculated as indicated in the Data and Methods section. The Pearson’s correlation coefficient between the annual mean Ta from ERA-interim and the AWS time series over the 2005–2016 period was 0.78, indicating that the reanalysis has a high skill in reproducing the annual Ta variability at QIC. As a reference, the mean annual ERA-interim bias corrected Ta at QIC is about −4.4 °C over the 1979–2005 period. The ensemble of 16 CMIP5 historical simulations and the reanalysis data are characterized by a common Ta warming rate of 0.14 °C/decade over the periods 1950–2005 and 1979–2016, respectively. It is worth noting that the CMIP5 interannual variability is substantially muted due to the cancellation of internal variability once multiple models are averaged to obtain the mean (hereafter labeled as ensemble). Future ensemble projections of Ta using RCP4.5 and RCP8.5 emission scenarios indicate substantial warming over the 2006–2100 period of about 0.25 °C/decade and 0.57 °C/decade, respectively. Those future scenarios suggest that the mean Ta at QIC summit will increase by approximately 2.4 °C and 5.4 °C, respectively, by the end of the 21^st^ century. This is consistent with results from previous studies over the tropical Andes^17,18^ using the older SRES scenario A2. Maybe more relevant in this context is the fact that under the RCP8.5 scenario Ta at QIC summit will surpass 0 °C by ~2060, while under the RCP4.5 scenario Ta will start to stabilize around −2 °C by ~2070.

### Long term evolution of the equilibrium-line altitude (ELA) at QIC

Since the date of the satellite images used to calculate the SLA (Table 1) typically falls toward the end of the hydrological dry season on QIC, the derived ELA is defined as representing the previous hydrologic year starting in September of the previous year and ending in the current August. Thus, the year label indicated in the x-axis in Fig. 2c corresponds to the August’s years.

**Figure 4:**
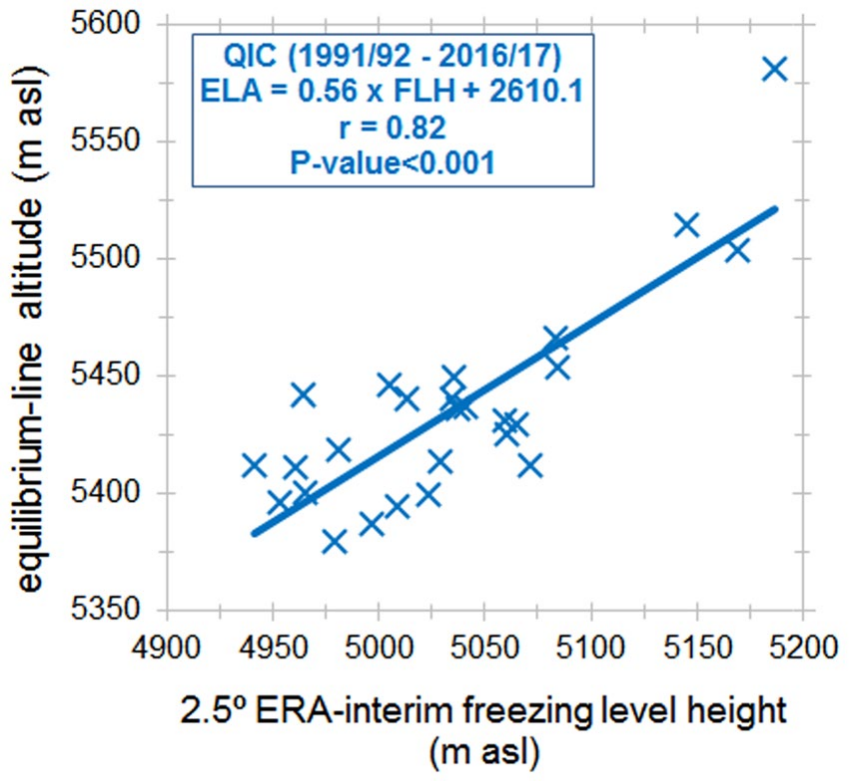
Scatter plot between annual FLH and annual ELA at QIC. FLH is calculated by interpolating bias-corrected air temperature (Ta) and geopotential height between 500 and 600 hPa from ERA-interim. The bias-corrected Ta was obtained by fitting reanalysis air temperature with observed Ta from an AWS at QIC summit. ELA data were obtained from LANDSAT images at the end of the dry season (see Table 1; median date 4 September). ELA and FLH were calculated for hydrologic years (September of previous calendar year to August). Pearson’s correlation coefficient (r) and p-value are indicated in the Figure.

The annual ELA was then compared with its corresponding FLH, which was calculated for the same hydrological year for the 1992–2017 period. Figure 4 presents the comparison between annual values of ELA and FLH obtained from ERA-interim reanalysis. There is a statistically significant linear relationship between the two variables, consistent with the similar linear relationship present over several other glaciers in the inner and outer tropical Andes^1^. The linear ELA – FLH relationship at QIC can be quantified as:

1 $${\rm{ELA}}\approx {\rm{0.56}}\times {\rm{FLH}}+{\rm{2610.1}}\,{\rm{m}},$$

with r = 0.82 and p-value < 0.001, over the 1991–2017 period. This relationship was subsequently used to project future changes in the ELA, using as input the FLH_atm_ and FLH_SST_ at QIC generated from CMIP5 historical and future scenarios. Calculated historical and future ELA’s from CMIP5 models were bias-corrected through comparison with the observed ELA during the period of overlap, 1992–2005 and 2006–2017 respectively.

### Future ELA projections at QIC

**Figure 5:**
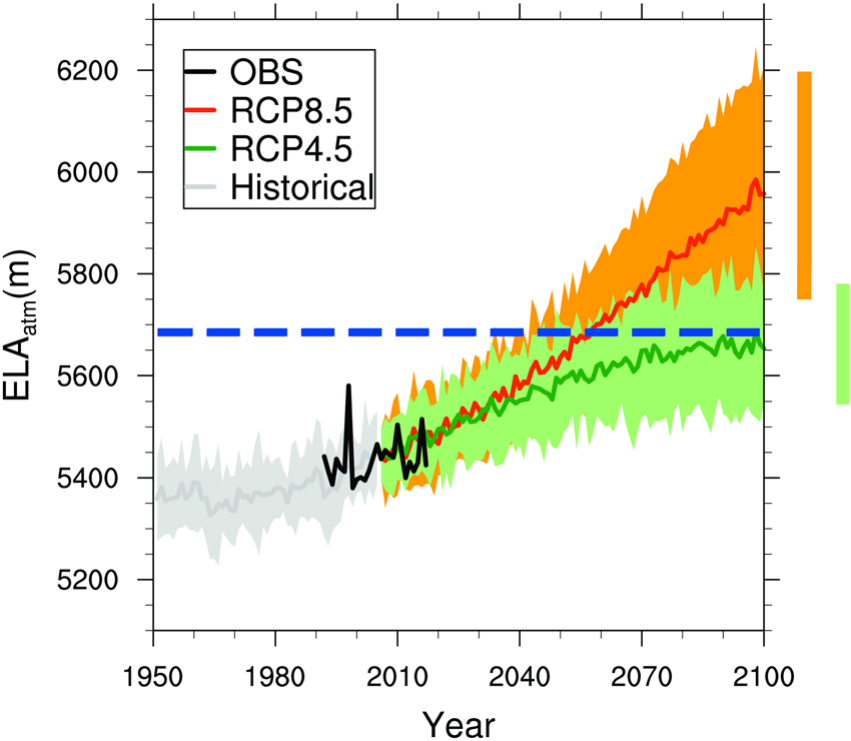
ELA projections at QIC. Equilibrium-line altitude (ELA) calculated using the freezing level height anomaly projections at QIC (FLH_atm_) from 16 CMIP5 models as input in equation (1). FLH_atm_ was obtained by interpolating Ta and Zg between 500 and 600 hPa pressure levels. The black curve (OBS) represents the observed ELA obtained from LANDSAT satellite images over the 1992–2017 period. Bias-correction was applied to Ta and ELA for each CMIP5 model, using the observed Ta from the AWS at QIC. The hydrological year Sept.-Aug. was used for calculations. The ensemble of historical (1950–2005) simulations is represented with the gray line, while CMIP5 RCP4.5 and RCP8.5 future projections (2006–2100) are represented by green and orange lines, respectively. The shading represents the corresponding 95% confidence intervals. The blue dashed line indicates the QIC summit altitude (~5680 m a.s.l.). The mean observed ELA over the 1992–2017 period was 5435 m a.s.l., and the mean ERA-interim ELA over the baseline 1979–2017 was 5416 m a.s.l. Likely ranges for ELA projections at QIC by the end of the 21st century are indicated by vertical bars.

Figure 5 presents observational, historical and future annual ELA projections for QIC. The mean ELA from observations was 5436 m a.s.l. over the 1992–2017 period, and the mean ELA value from ERA-interim over the baseline 1979–2005 was 5416 m a.s.l. (data not shown). The increase in the ELA is +16.3 m, +13.6 m, +24.5 m and +58.4 m/decade, for reanalysis (1980–2017), historical (1950–2005), RCP4.5 and RCP8.5 (2006–2100), respectively. Based on the multi-model mean estimate, the ELA will remain below the QIC summit until the end of the 21^st^ century in the RCP4.5 scenario, although some CMIP5 models are projecting a future ELA that is higher than the QIC summit.

For the RCP8.5 scenario the changes at QIC are going to be more profound and occur much earlier than in the case of the RCP4.5 scenario. By the middle of the century the ensemble mean ELA is projected to reach the QIC summit, turning all of QIC into an ablation zone. A very important point is that the QIC summit at 5680 m a.s.l. will of course be continuously lowered, once the ice cap increasingly thins from its current thickness of approximately 170 m^44^. Hence it will be exposed to higher temperatures at lower elevation (elevation feedback), as well as increasingly to edge effects and warm air advection from surrounding exposed bare rock areas as the ice cap shrinks in size (edge effects feedback). These feedbacks are not accounted for in our analysis, suggesting that our results likely err on the conservative side and that the ELA may in fact reach the QIC summit earlier than projected in our analysis. Finally, the RCP8.5 Ta projections at QIC suggest that at the end of the 21^st^ century temperature will have increased by about 5.4 °C (see Fig. 3). This implies that the ensemble mean yearly average Ta at the QIC summit will be near +1 °C. Precipitation events at this temperature threshold will likely be divided in snow, rain and mixed precipitation in similar proportion^48^.

How long it will actually take for QIC to completely disappear is a different question and beyond the scope of this study, but it is evident that runoff from QIC during the dry season will eventually decrease significantly. Until then, melt water from the receding ice cap may for a period of time enhance the glacial melt water contribution^35,49^.

### On the relationship between FLH at QIC and tropical SST

As pointed out in several studies, a linear relationship exists between tropical SST and tropical FLH^26,27,41,42^, including over the Andes^8,9,24,50–53^. This relationship can be exploited to calculate how the FLH over QIC will change in the future, using future projections of tropical SST, and assuming that the FLH-SST relationship remains stable over time (i.e. no change in lapse rate). Here we calculate the FLH at QIC using reanalysis products, and linearly relate it with tropical SST^9^, to assess future FLH changes under such a fixed lapse-rate scenario. These FLH estimates, henceforth referred to as FLH_SST_ can then be compared with future FLH changes simulated by coupled ocean-atmosphere CMIP5 models (FLH_atm_), with the difference being a measure of future adjustment in the lapse rate and thus of EDW. Paleoclimate studies have documented how this tropical lapse rate, linking SST with the snowline in tropical mountain regions, has changed in the past^41^ and they have been used to constrain future amplified high-elevation-warming in tropical mountain regions^42^. Here we apply the same methodology to the QIC.

**Figure 6:**
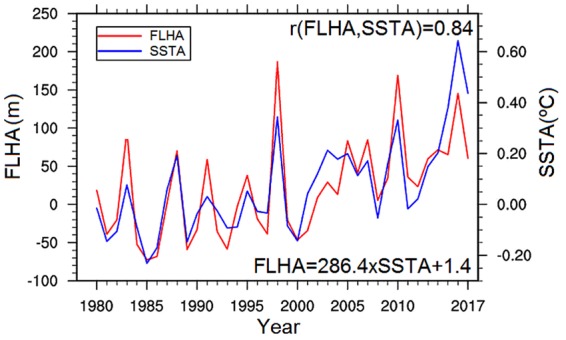
FLH – SST linear relationship. Tropical SST forcing of FLH at QIC during 1980–2017 (hydrologic years). Annual FLH anomalies (FLHA) at QIC were calculated by interpolating bias-corrected air temperature (Ta) and geopotential height (Zg) from ERA-interim reanalysis, between 500 and 600 hPa pressure levels at QIC summit location (red line). The bias-corrected Ta was obtained by fitting the ERA-interim Ta product with observed Ta data from an AWS at QIC summit. The annual mean tropical SST anomalies (spatially averaged across 28.75°N to 28.75°S) were calculated from ERSST data (blue line). Anomalies were calculated using the baseline 1979–2005 period. The linear relationship between FLHA and tropical SSTA can be expressed as FLHA = 286.4 × SSTA + 1.4m, with r = 0.84 and p-value < 0.001.

The hydrologic year FLH at QIC was calculated from ERA-interim reanalysis products (as indicated previously in the Data and Methods section), and compared with tropical SST (spatially averaged from 28.75°N to 28.75°S). The resulting linear relationship between annual anomalies of FLH (FLHA) at QIC and tropical SSTA (°C) (Fig. 6) over the 1980–2017 period (38 hydrologic years), is expressed as:

2 $${{\rm{FLHA}}}_{{\rm{SST}}}=286.4\times {\rm{SSTA}}+1.4\,{\rm{m}},$$

with, r = 0.84 and p-value < 0.001. This statistical relationship quantifies how tropical SST relates to atmospheric temperature at QIC under present conditions, explaining 71% of the total variance in FLHA_SST_. Projecting this relationship into the future, however, will underestimate the actual rise of the FLH, given multiple elevation-dependent feedbacks that will likely lead to enhanced future warming at higher elevations. Indeed, the increase in atmospheric water vapor will likely result in enhanced release of latent heat during tropical convection and condensation, thereby warming the tropical mid- and upper troposphere. In addition, the increase in water vapor in the upper troposphere will exert a stronger radiative effect, given its lower initial concentration, thereby contributing to a stronger warming at higher altitudes^20^. This enhanced longwave downwelling radiation feedback is likely a significant driver of the FLH increase, but several other feedbacks affecting elevated regions like QIC, such as clouds, albedo, and aerosols may also play a role^20,21^.

### Elevation-dependent warming (EDW) quantification at QIC

To verify and quantify how much the EDW will affect QIC in the 21^st^ century, we present a comparison between the ELA_SST_ and ELA_atm_ at QIC, both bias-corrected and calculated based on equation (1), but using as input the FLH_SST_ from equation (2), and FLH_atm_ derived by interpolating Ta and Zg, respectively, from 16 CMIP5 models. The ERSST and ERA-interim reanalysis products were used as a control case.

**Figure 7:**
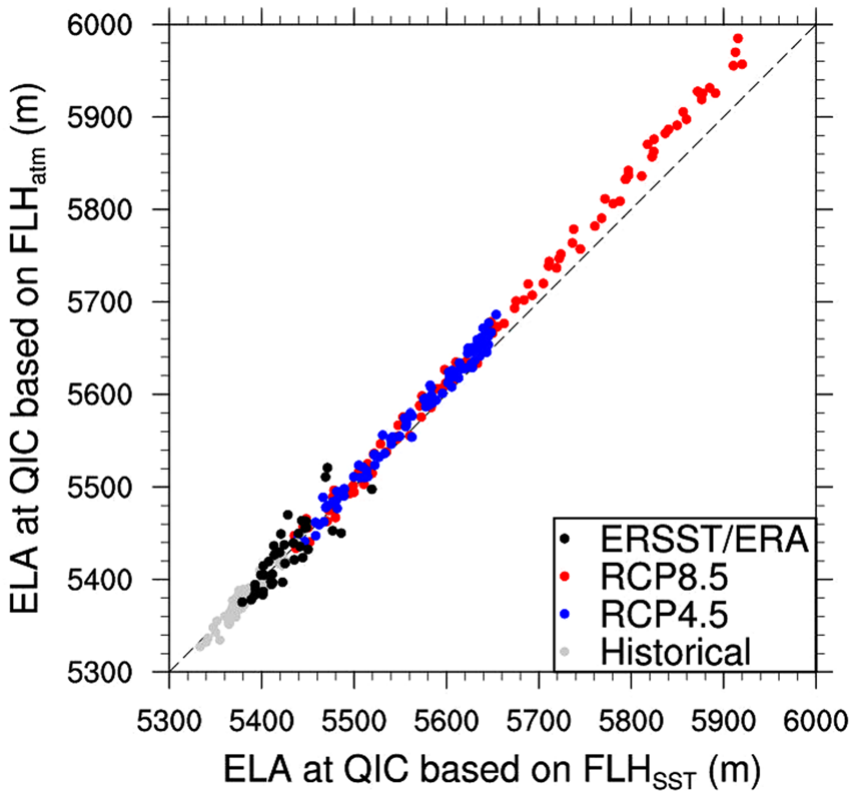
ELA_atm_ vs ELA_SST_ comparison. Annual mean ELA derived from FLH at QIC calculated by linear regression (equation 1) with annual mean tropical SST from ERSST dataset as predictor (FLH_SST_ using equation 2), compared with ELA derived by interpolating air temperature (Ta) and geopotential height (Zg) from ERA-interim reanalysis between 500 and 600 hPa levels (FLH_atm_) (black dots). The same approach is applied to the ensemble mean of annual FLH_SST_ and FLH_atm_ obtained from 16 CMIP5 models for Historical (gray dots), RCP4.5 (blue dots) and RCP8.5 (red dots) scenarios. Historical and future simulations were analyzed over the periods 1951–2005 and 2006–2100, respectively. Dashed line represents the 1:1 line. A bias-correction was applied to Ta and ELA, using data from the AWS at QIC summit elevation (5680 m a.s.l.) and estimated highest annual snowline altitude from Landsat images, respectively.

In Fig. 7, the control case results (black dots) are consistent with the historical comparison between the ELA_SST_ and ELA_atm_ ensembles from 16 CMIP5 historical simulations (gray dots). Future projections of ELA_atm_ ensembles, based on 16 CMIP5 RCP4.5 (blue dots) and RCP8.5 (red dots) simulations, however, are not following the expected ELA increase inferred from the ELA_SST_ model. For instance, in the RCP4.5 scenario, the increase in the ELA_SST_ ensemble is about 22.8 ± 0.74 m/decade; less (albeit not significantly) than the ensemble ELA_atm_ increase of 24.5 ± 0.94 m/decade. In the case of the scenario RCP8.5, this difference in ELA trends is significant (p < 0.05), with 52.9 ± 1.04 m/decade and 58.4 ± 1.45 m/decade for ELA_SST_ and ELA_atm_, respectively. This implies that CMIP5 simulations with RCP4.5 radiative forcing at the end of the 21^st^ century generate an insignificant additional rise of the ensemble mean ELA at QIC of about 1.72 m/decade when compared to the tropical SST forcing, while the RCP8.5 radiative forcing generates an additional, statistically significant ELA rise of about 5.5 m/decade. This additional ELA rise can be understood as an EDW response, resulting from feedbacks that effectively lead to a flattening of the tropical lapse rate. Thus, for a more intense anthropogenic radiative forcing scenario, the EDW effect will increase. This analysis verifies and quantifies the projected EDW effect over the QIC environment in response to future changes in radiative forcing.

The difference between the ensemble mean ELA_atm_ and ELA_SST_ of about 5.5 m/decade for the RCP8.5 scenario implies that by 2055, the ensemble ELA_atm_ will reach the QIC summit level, while the ELA_SST_ ensemble will still be ~30 m below the QIC summit. In other words, the ELA_atm_ will reach the QIC summit about 12 years earlier than the ELA_SST_. Note that these calculations were done for the ensemble of 16 CMIP models, and that results for individual CMIP5 models vary considerably.

## Discussion and Conclusions

CMIP5 model simulations of future temperature changes were applied to study the impacts of climate change over the Quelccaya Ice Cap region, assessing the relationship between ELA and FLH and how these variables relate to tropical SST forcing in the past and the future. We did not consider the influence of potential future changes in ENSO behavior on QIC climate, which would be relevant for understanding future changes in interannual variability and will be included in future work, but present-day ENSO variability over the period for which the model was built is implicitly included.

Here we relied on air temperature (and FLH) from global products (reanalysis and CMIP5) since a comparison with *in-situ* data from our AWS showed that these products very faithfully reproduce temperature conditions on QIC once a bias–correction is applied, consistent with earlier studies^37,54^. Hence while the application of free-tropospheric air temperature in our model did not require a more sophisticated statistical downscaling method^53,54^, a comparative study between our results and other downscaling products (e.g., CORDEX) would be a worthwhile follow-up study.

One important outcome of this study is that the ELA will strongly be affected by feedback mechanisms that accelerate the warming at high elevations, most noticeable in the RCP8.5 scenario. QIC is likely to completely loose its accumulation zone before the end of the 21^st^ century. The critical time when the ensemble RCP8.5 simulated ELA will reach the QIC summit is around 2055, concomitant with RCP8.5 annual average Ta at QIC summit rising to approximately −1.8 °C. From that point forward, the ELA will remain above the ice cap’s highest elevation, leaving the entire ice cap exposed to a continuously negative surface mass balance. However, other factors, not considered here, can change this timing, such as changes in the amount or seasonality of precipitation. Since changes in precipitation are a more complex issue to assess, it will be dealt with in a separate analysis. As far as precipitation phase is concerned, Ta = −1 °C is the critical threshold where precipitation starts to change phase in the tropical Andes^48^, leading to a decrease in snowfall and increase in mixed precipitation. Based on our results, this threshold will be reached around 2070 for a few models in the RCP4.5 scenario and the ensemble mean Ta for the RCP8.5 scenario.

Here we have focused on the influence of tropical SST on FLH over QIC, since tropical SST provide the dominant first-order control on FLH and snowline altitude throughout tropical mountain regions^9,25–28,41,42^. Indeed tropical SSTA explain more than 70% of the total variance in the FLH over QIC on interannual timescales. While other factors such as land surface feedbacks, tropical convection over the Amazon basin and interactions with extratropical air masses can also influence the FLH over Quelccaya on interannual timescales^13,22^, their influence is rather limited and they are by no means independent from tropical SST.

Although the ELA is closely correlated with the FLH_atm_ and previous studies have documented that this relationship holds throughout the tropical Andes^1,25^, this prediction of the ELA could potentially be further improved by applying a multivariate model including other atmospheric variables, such as precipitation and wind field. We have, however, opted not to consider such variables, given the large uncertainties in future projections of precipitation over the Andes. In our view the added values from inclusion of these variables is offset by much larger uncertainties in the future projections. That said, the presented uncertainty ranges in the timing of ELA reaching the summit should themselves be considered conservative estimates.

More research is needed to further clarify the nature of the feedbacks that lead to the anticipated elevation-dependent warming on QIC. In addition, it is critical to better quantify elevation feedbacks, edge effects, and the impacts of changing precipitation phase with higher FLH on QIC’s surface mass balance. As these aspects will promote an even faster demise of the ice cap, our projections are likely conservative estimates.
